# Development of a web-based platform for the semi-structured record of the psychiatric interview during clinical practice: an opportunity to impact research and improve health care

**DOI:** 10.1192/j.eurpsy.2022.1954

**Published:** 2022-09-01

**Authors:** D. Vasquez, C. Velez

**Affiliations:** 1 Universidad de Antioquia, Grupo De Neurociencias De Antioquia, Medellin, Colombia; 2 Montgomery College, Mathematics And Data Science Department, Rockville, United States of America

**Keywords:** Medical Record, Big Data, Web-based platform, Clinical research

## Abstract

**Introduction:**

Managing healthcare data is a major challenge for today’s medicine. The use of artificial intelligence and big data tools has allowed solving questions related to this topic. However, the wide heterogeneity in the psychiatric consultation record makes the retrospective analysis of these data limited due to a lack of information or differences between specialists.

**Objectives:**

We aim to develop a platform that allows the structured record of medical care data (based on dementia) while maintaining flexibility and format for its usefulness during clinical practice in psychiatry.

**Methods:**

We developed a web-based platform for the structured and semi-structured record of psychiatric evaluation. The instrument is diagnosed-oriented (for our version we used dementia). We used Core outcome sets and expert opinion to identify the relevant outcomes for the attention.

**Results:**

A web-based platform is presented for the care of people with suspected dementia at different levels of care designed with the potential to record information of interest in research but also of clinical utility for closer follow-up.

**Conclusions:**

This strategy allows developing the proposal towards other pathologies of interest. Also, with the integration of recommendation algorithms, a monitoring and recommendation system could be achieved to promote knowledge of psychiatric illness from routine practice. This proposal intends to have an impact by increasing the quality of care, reducing care times, and providing better approaches from primary care systems.

**Disclosure:**

No significant relationships.

